# A Method for Estimating Longitudinal Change in Motor Skill from Individualized Functional-Connectivity Measures

**DOI:** 10.3390/s22249857

**Published:** 2022-12-15

**Authors:** Nader Riahi, Ryan D’Arcy, Carlo Menon

**Affiliations:** 1 Schools of Engineering Science, Simon Fraser University, Burnaby, BC V5A 1S6, Canada; 2 DM Centre for Brain Health, Department of Radiology, University of British Columbia, Vancouver, BC V6T 1Z4, Canada; 3 HealthTech Connex, Surrey, BC V3V 0E8, Canada; 4 Biomedical and Mobile Health Technology Laboratory, Department of Health Sciences and Technology, ETH Zurich, 8008 Zurich, Switzerland

**Keywords:** motor skill assessment, EEG sensors, resting state functional connectivity, phase lag index, partial least squares correlation and regression

## Abstract

Pragmatic, objective, and accurate motor assessment tools could facilitate more frequent appraisal of longitudinal change in motor function and subsequent development of personalized therapeutic strategies. Brain functional connectivity (FC) has shown promise as an objective neurophysiological measure for this purpose. The involvement of different brain networks, along with differences across subjects due to age or existing capabilities, motivates an individualized approach towards the evaluation of FC. We advocate the use of EEG-based resting-state FC (rsFC) measures to address the pragmatic requirements. Pertaining to appraisal of accuracy, we suggest using the acquisition of motor skill by healthy individuals that could be quantified at small incremental change. Computer-based tracing tasks are a good candidate in this regard when using spatial error in tracing as an objective measure of skill. This work investigates the application of an individualized method that utilizes Partial Least Squares analysis to estimate the longitudinal change in tracing error from changes in rsFC. Longitudinal data from participants yielded an average accuracy of 98% (standard deviation of 1.2%) in estimating tracing error. The results show potential for an accurate individualized motor assessment tool that reduces the dependence on the expertise and availability of trained examiners, thereby facilitating more frequent appraisal of function and development of personalized training programs.

## 1. Introduction

There is widespread interest in using objective neurophysiological measures for assessment and monitoring of longitudinal change in motor function [1,2,3,4,5]. Addressing the technical and pragmatic challenges associated with estimating motor function might help bring about more frequent assessments and facilitate the development of a personalized training strategy. Pragmatic considerations should include cost, availability, and duration of the assessment. Device portability and elimination or reduction of required physical tasks can help in this regard. With respect to technical considerations, the assessment tool must achieve comparable accuracy to conventional protocols used for evaluating motor function and require minimal expertise to carry out the measurements.

Execution of motor tasks involves synchronized activities of broad areas of the brain [6,7]. The selection of relevant neurophysiological measures should therefore allow for the evaluation of wide-ranging network interactions. Brain FC is a good candidate in this regard. FC is generally quantified in terms of correlation in activities of different brain areas [8]. Empirical research with electroencephalography (EEG) has shown that engagement and communication between brain regions are facilitated through neural synchronization at different frequencies [9,10], and that the synchronization parameters can be used as metrics to quantify FC between associated brain areas [11]. The high temporal resolution of EEG systems is favourable towards accurate measurement of synchronization (coherence) between different regions of the brain and is why EEG systems are commonly used in studies where coherence is selected as a measure of FC. This dynamic change in FC is at play for both task-based and resting state activities of the brain [12]. Prior studies have investigated the changes in rsFC of stroke survivors during acute and sub-acute phases as a consequence of rehabilitation [13,14] and its contribution towards prediction of future outcome and stratification [15]. Resting state FC has also been used to investigate the global network interactions and their influence on motor performance in healthy individuals [16]. The resting state analysis eliminates the need for execution of physical tasks and can potentially result in a shorter assessment time. When considering the relative low cost and portable nature of EEG systems, the use of rsFC for estimating motor function can meet the pragmatic requirements of the assessment tool.

Pertaining to technical considerations and specifically the accuracy of assessment, rsFC has shown promise in estimating motor function [17,18]. In these studies, coherence at different canonical frequency bands were used as a measure of rsFC and Fugl-Meyer assessment scores were used as a measure of motor impairment in stroke survivors. Partial Least Squares (PLS) algorithms were applied to estimate motor function from rsFC measures. PLS is a multivariate statistical method that uses singular value decomposition to project the covariance of input variables onto the latent space for correlation analysis and generation of regression models for the prediction of dependant variables [19]. PLS is particularly useful when working with very large number of independent variables and a small number of observations [20]. Although these studies showed good results in estimating motor function, the accuracy of the algorithms was evaluated against subjective measures (Fugl-Meyer scores) that quantify relatively large changes in motor function [21]. As an example, the hand movement from ipsilateral ear to contralateral knee for each of the shoulder, elbow, and the forearm was assessed with score levels quantified at 0 (can not be performed), 1 (partial motion), and 2 (full motion) [22]. To gain a better understanding of the estimation accuracy of these algorithms, their performance needs to be evaluated against a more objective measure of behavior that can be quantified at smaller incremental change as compared with conventional assessment protocols. Specific to motor function, longitudinal acquisition of a skill by healthy individuals through physical training is a good candidate for this purpose. Computer-based tracing tasks, where the deviation from graphical tracks is used for the assessment of tracing skill, are an example of such objective measures. The ability of the algorithms to accurately estimate modest changes in motor skill may prove useful in addressing the technical requirements of the assessment tool.

Longitudinal motor learning studies with healthy participants using functional magnetic resonance imaging showed that some areas of the brain exhibited a transient change in FC while other areas showed a more lasting change towards consolidation and long-term retention [23,24,25]. This persistent change in FC has the potential for quantifying, and subsequently estimating the change in motor skill. Prior work using EEG modality showed correlation between rsFC and motor learning [26], with several studies focusing on the ability of generalized global configuration of FC to estimate skill acquisition in healthy participants [27,28,29,30,31,32]. The authors showed interaction between a wide range of brain networks at different synchronization frequencies for motor learning. Involvement of different networks and frequencies, along with differences across subjects due to age or existing capabilities, motivates an individualized approach towards the evaluation of correlating FC measures. As such, we explored a longitudinal motor skill training program involving a computer-based tracing task, in which healthy participants used a computer mouse with their non-dominant hand to trace a predetermined and non-trivial pattern on a computer screen. The aim was to improve motor skill by attempting to reduce tracing error over a period of six to eight training sessions spread over as many days. Tracing error was used as an objective measure of behavioral performance that could be quantified at small incremental changes. Resting state EEG data were collected before and after each training session and used for the evaluation of rsFC. We then investigated the accuracy of an individualized PLS method in estimating longitudinal change in skill from changes in rsFC.

## 2. Materials and Methods

### 2.1. Study Design

#### 2.1.1. Workflow

**Figure 1:**
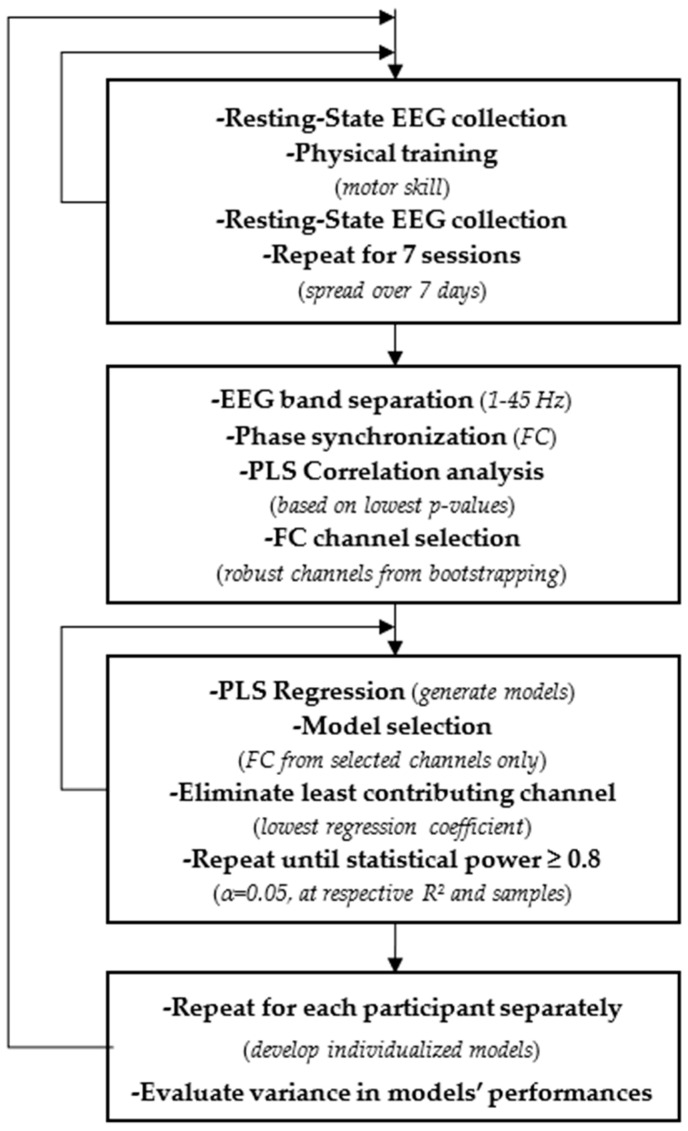
Experimental workflow for data collection and analysis.

Figure 1 shows the experimental workflow for data collection and analysis. Tracing errors during physical training were used to appraise the change in acquired motor skill.

#### 2.1.2. Setup

We used a 32-electrode gel-based EEG cap (g.Nautilus, g.tec medical engineering, Austria) operating at a sampling rate of 250 Hz, and OpenVibe v2.2 for data acquisition and storage. The reference electrode was placed on the right earlobe, with the ground electrode located midway between FZ and FPZ. We applied enough conductive gel to maintain contact impedance below 30 K-Ohm. Recorded EEG data were imported into MATLAB-7.8.0 (MathWorks Inc., Natick, MA, USA) for signal processing and analysis.

**Figure 2:**
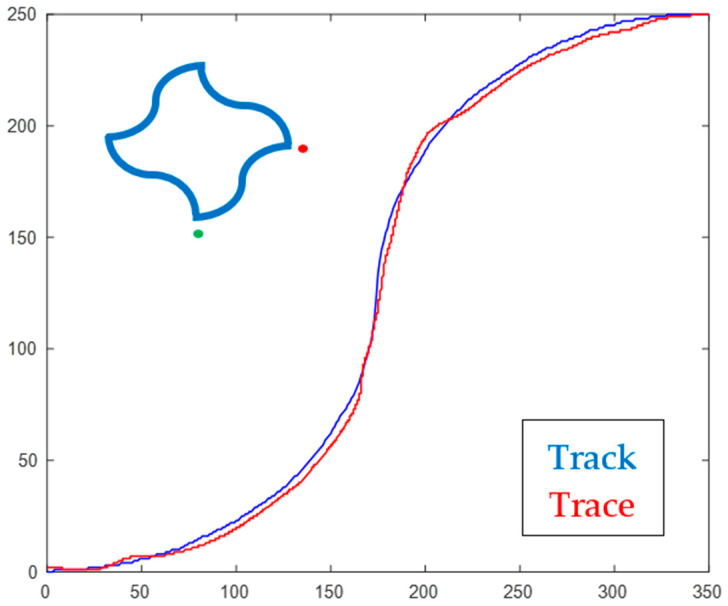
Participants’ tracing trajectory over a track section. The position–error is the total area between the Trace (red) and Track (blue) section. The inset at the top-left corner shows the complete track pattern. The green and red dots identify the active track to be traced. Axes are in units of screen pixels. Reproduced with permission from [33].

Python 3.7 was used to create our experimental track patterns on a computer screen. We opted for elliptical trajectories instead of straight lines [31] to increase the degree of difficulty for tracing tasks. The track was constructed from eight quarter-ellipses that were arranged to form a four-section curved-pattern, as shown in the inset (top-left corner) of Figure 2. In this figure, the green dot represents the starting point for the placement of the mouse pointer before tracing the corresponding track towards the red dot. The selection of the active track section was controlled through the program, as explained later in the protocol. We adjusted the pointer speed so that the distance between opposing tips of the track pattern corresponded to approximately 35 cm of mouse travel across the torso (*x*-axis: left to right) and 25 cm away from the torso (*y*-axis: top to bottom). The rational was to promote large enough physical movements to engage multiple arm joints without the need to move the torso. We also disabled the driver option that caused a non-linear relation between the pointer’s speed and the mouse acceleration. The objective was to maintain relational consistency between the mouse and pointer coordinate systems. The horizontal area in physical space was thus represented by pixels on the computer screen, and time was measured through the computer’s real-time clock. Total position–error while tracing a track section was determined by the area (in pixels) between the actual pointer trajectory (Trace) and the desired track path (Track), as shown in Figure 2.

#### 2.1.3. Participants

Seven healthy right-handed participants, HP1 through to HP7 (Mean Age = 38.7, SD = 21.5, 3 females), volunteered for the research study. Participants had no known neurological conditions, artifact-inducing implants, or physical conditions that would exclude them from the study. The Research Ethics Board of Simon Fraser University approved the protocol for this study, and all participants signed informed written consent forms.

#### 2.1.4. Protocol

Every participant completed a longitudinal experiment that included seven sessions, limited to a single session per day over two weeks. Each session consisted of four phases. There were no breaks in between or during each phase unless the participant specifically asked for one due to fatigue.

- Phase-1: The pre-training 5-min resting state EEG data collection. Participants were asked to sit comfortably upright with their feet flat on the floor, still and quiet with their eyes closed, but awake. Participants were notified of the start of EEG recording.
- Phase-2: An 8-trial test, with each trial including a tracing task with the right hand (dominant hand). Data from Phase-2 were collected but not used in this study.
- Phase-3: 90-trial training, with each trial including a tracing task with the left hand (non-dominant hand). These trials were over a randomly selected section of track and direction of tracing. Participants were asked to move the mouse on the table using only their arm and not their torso. Participants were prompted to move the mouse-pointer to the vertex identified by the green dot and instructed to trace the track towards the vertex with the red dot (Figure 2). They were asked to trace quickly and accurately, without compromising one for the other. To complete each trial, participants had to keep the pointer at the destination vertex for one second. This would penalize performance indicators when moving too fast to stop at the destination vertex.
- Phase-4: Post-training 5-min resting state EEG data collection.

Each session lasted between 70 to 90 min depending on EEG setup time and the participants’ tracing speed during Phase-3.

### 2.2. Tracing Performance

Position–error between the participants’ tracing trajectory and the intended track pattern was selected as one of the performance indicators. Time taken to trace each section (trial) was selected as another indicator. Total position–error during a trial was determined by the area between the trace and track trajectories (Figure 2). To discourage participants’ attempt to reduce position–error by tracing slower, we used each trial time as a penalizing (multiplication) factor to inflate the respective position–error. Conversely, to discourage participants’ attempt to reduce trial time by moving the mouse too quickly to stop at the destination vertex, we accumulated positional offsets from the track endpoint until the pointer came to rest at the destination vertex.

We used both accumulated position–error as well as the accumulated product of position–error and its corresponding tracing time from each trial, as two separate indictors of tracing performance. Changes in motor skill between sessions were reflected in variations in the magnitude of one or both of these measures. The selection of two metrics was our attempt in addressing the differences in participants that were more focused on tracing error rather than the tracing speed (or vice versa). We speculate that these may involve different interacting brain regions. Participants were updated on their tracing performance after each training session. Position–error was measured in units of pixels-squared, representing the area between the track and trace trajectories, and was converted to spatial units of squared-centimetres (cm^2^) based on an estimated coverage of 0.25 mm^2^ per pixel. The product of position–error and its associated tracing time was measured in units of cm^2^ s.

Each training session consisted of 90 tracing trials, generating 90 intermediate performance values. We used two different approaches to produce a measure of tracing performance for each session. First, a single value corresponding to the median of all 90 trials (single-median option), and second, the median of the first 30 trials as a measure of performance before training, and the median of the last 30 trials as a measure of performance after training (dual-median option). The two measures allowed for separate evaluation of consolidated and short-term learning.

### 2.3. EEG Data Processing

Longitudinal EEG data from each participant were processed independently of the other participants, making this an individualized assessment rather than a cross-sectional or inter-participant analysis. We used an EEG sampling frequency of 250 Hz and a frontend finite impulse response bandpass filter of 1–45 Hz. We defined five canonical frequency bands specified as Delta (1–4 Hz), Theta (4–8 Hz), Alpha (8–15 Hz), Beta (15–30 Hz), and Gamma (30–45 Hz) for our second stage filters and band separation. We further divided each band into low, medium, and high sub-bands, resulting in 15 distinct center-frequencies and 15 different Morlet wavelets for bandpass filtering [34]. Center-frequencies were approximately one bandwidth apart. We used coherence as a measure of functional connectivity. In general, evaluation of coherence could involve the use of both amplitude and phase. Spectral coherence is an example of this. Excluding the power is commonly referred to as synchronization. For the sake of simplicity, we use the term coherence and synchronization interchangeably, but clarify that spectral coherence involves the evaluation of phase synchronization that is modulated by power. We computed the instantaneous coherence through five different algorithms, namely Phase-Clustering, Spectral Coherence, Imaginary part of Coherence, Phase Lag Index (PLI), and weighted PLI [34]. Coherence was evaluated for every combination of electrode-pairs (496 channels) at each of the 15 center frequencies. Resulting coherence measures from each algorithm were then separately averaged over 1-s non-overlapping epochs, generating a total of 300 samples for each channel and at each frequency. For the sake of consistency with our prior study with stroke survivors [18], we used only a 2-min section (120 samples), starting at an offset of 30-s from the beginning of EEG data for PLS analysis. The 30-s offset was selected to allow the participants to settle into a resting state, in particular for post-training EEG collections where the participants may have still been thinking about their tracing tasks. For each of the five coherence evaluation algorithms, we used the maximum sample value (peak-detected) out of the 120-sample window to generate a single connectivity index for each channel. Both single-median and dual-median behavioral measures were used for correlation analysis with the connectivity indices. We selected the median of all 90 trials for each session as the behavior data (tracing performance) associated with that session for the single-median option. These were used for correlation analysis with connectivity indices from both the pre- and post-training EEG data separately. For the dual-median option, a correlation analysis was carried out between the median of the first 30 trials and the connectivity indices from the pre-training EEG, and the median of the last 30 trials with that of post-training EEG.

### 2.4. PLS Analysis

PLS takes advantage of principal component analysis to address the potential collinearity that might exist between a large number of independent variables in the feature space. Similar approaches such as principal component regression try to achieve this by first finding the orthogonal components of the independent variables and then selecting the first few components to develop a regression model for predicting the dependant variable. Although these components can optimally explain the variance in the independent variables, there is no guarantee that they are the most relevant predictors of the dependant variable [20]. PLS regression, however, finds the principal components of the covariance of the independent and dependant variables and builds a regression model through an iterative deflation process.

We used PLS Correlation (PLSC) to identify the most robust channels and frequencies that contributed towards correlation between the connectivity indices and the selected measure of tracing performance [35]. We selected the options with the lowest *p*-values for this purpose. We repeated the process 50 times for each participant and selected the channels that were consistently present in over 80% of the repetitions and at the same level of robustness. We used permutation for quantifying the *p*-values and bootstrap to test for robustness. The resulting connectivity indices for the identified channels and frequencies were then used in PLS-Regression (PLSR) to produce a model for estimating the corresponding tracing performance. In brief, singular value decomposition of the covariance matrix generates the first set of latent vectors for the dependant and independent variables that explain the largest variance. The first regression coefficient in the latent space was computed from these vectors. The vectors were also used to compute an estimate of the input matrices that were subsequently used to deflate them. The process was then repeated with the deflated matrices until full deflation or stopped after finding a reduced number of predictive latent variables. The regression coefficients were then brought back into the feature space to construct a model for estimating the dependant variable from independent variables [20,35].

The estimation accuracy was examined through a cross-validated leave-one-out approach over the seven training sessions and quantified by the root-mean-square-error (RMSE) in estimation. The estimation error was presented as a percentage of the average tracing error obtained from the selected single- or dual-median option. The ratio of estimation RMSE over average tracing error allowed for comparison between estimation accuracy from different tracing performance measures obtained through position or position–time error. Data from participants with multiple combinations of contributing channels and frequencies were passed through iterative PLSR analysis. The goal was to find the specific combination that resulted in the smallest RMSE in estimating the tracing performance with the lowest number of contributing channels that resulted in a statistical power of 0.8 at α = 0.05 for the number of available samples for each participant.

## 3. Results

### 3.1. Data Collection

We carried out 53 training sessions, collected over 500 min of EEG data and 4700 tracing trials from seven participants. Data from four sessions were discarded due to external noise and technical recording issues. Except for HP1 and HP2, all other participants had 7 training sessions with 70 min of EEG data and 630 tracing performance measurements for each participant. Participant HP2 had to stop after 6 sessions and participant HP1 volunteered for 8 sessions.

### 3.2. Tracing Performance

**Figure 3:**
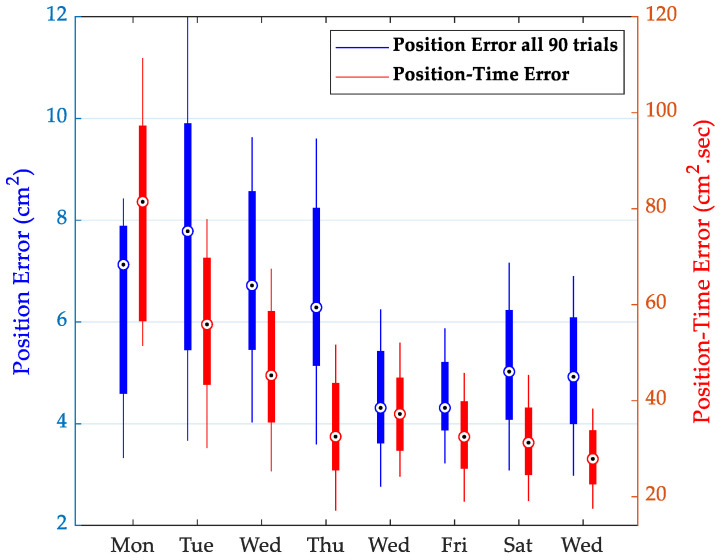
Longitudinal tracing performance for HP1 during 8 sessions of physical training program with 90 trials in each session. Performance is in terms of position error (blue bars) and product of position error and time (red bars). The bullseye indicates the median value. Corresponding results from the first and last 30 trials were similar in nature and were excluded for the sake of clarity.

**Figure 4:**
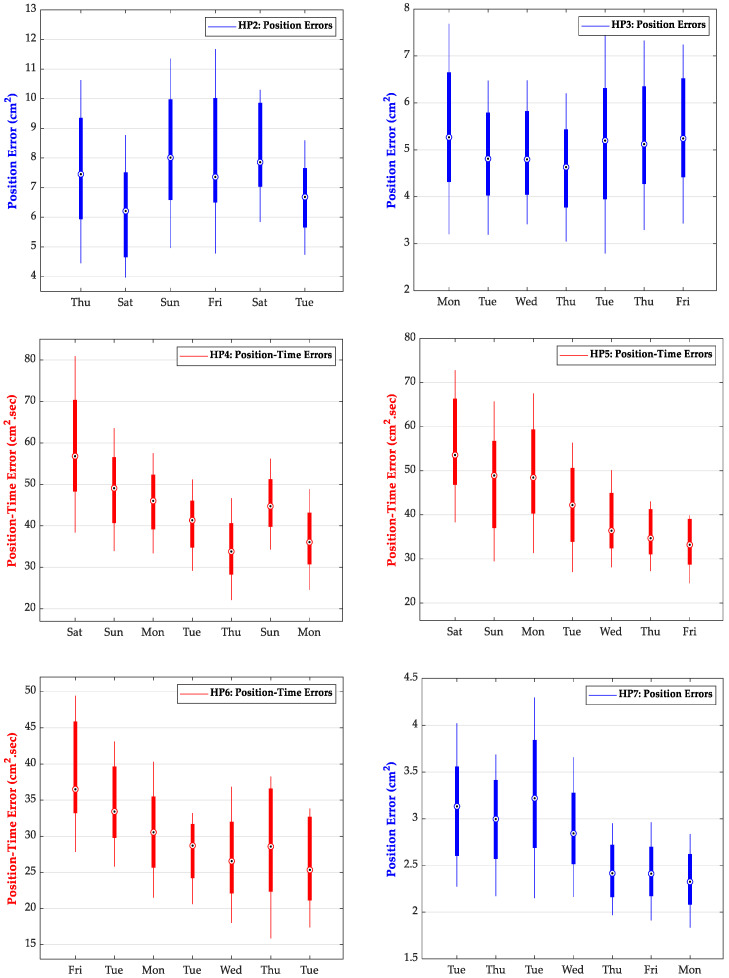
Longitudinal tracing performance in terms of Position Error (blue bars) or Position–Time Error (red bars) for participants HP2 to HP7. The selection of performance measure is based on the final PLS analysis, as shown in Table 1.

Figure 3 shows the results of longitudinal training for participant HP1 with position error (blue bars) and position–time error (red bars) from all 90 tracing trials. The bullseye represents the median value. The results for the first and last 30 tracing trials were similar in nature and were omitted for the sake of clarity and space. Note that a change in one metric without a corresponding change in the other could be used to identify and isolate the active metric. For example, a participant that focuses primarily on the position error may achieve a change in this measure of performance by slowing the speed of tracing. This could potentially appear as very little overall change in position–time measures. The opposite situation would correspond to a participant that focuses primarily on speed with less attention to positional error. These activities may involve different groups of interacting brain areas, resulting in different models for predicting the tracing performance. Figure 4 shows the longitudinal training results for the remaining participants HP2 to HP7 but is limited to the final selection of the position or position–time errors after PLSR analysis, as shown in Table 1.

### 3.3. PLS Analysis

Synchronization values using the PLI algorithm, averaged over 1-s non-overlapping epochs, and peak-detected across a 2-min EEG interval at a start-offset of 30-s, resulted in strong correlation (*p* < 0.05 uncorrected) between the tracing performance and connectivity indices. For most participants, PLSC analysis generated multiple combinations of promising correlation between rsFC extracted from pre- or post-training EEG data and different tracing performance measures. We used bootstrapping to identify the most robust channels and center frequencies that contributed towards the correlation with tracing performance. PLSR analysis was constrained to these channels and frequencies. We generated a regression model for each combination and applied cross-validated leave-one-out approach to evaluate the estimation RMSE, which was subsequently used to represent the estimation accuracy. We then selected the model with the lowest RMSE as the best performing estimator of the change in motor skill for that participant. RMSE was presented as a percentage of the average tracing error for the respective participant. This allowed for comparison between estimation accuracy from different tracing performance measures obtained through position or position–time error.

Regression coefficients represent the contribution of corresponding channels (predictors) at the identified frequency bands towards estimating the tracing performance (behavior) from rsFC indices. The small number of training sessions (samples) for each participant has a negative impact on the statistical power of our analysis. We therefore aimed to reduce the number of predictors to counter the effects of our small sample size. The aim was to reduce the channel count to the maximum number of predictors that resulted in a statistical power of greater than 0.8 at α = 0.05, given the R^2^ and the number of samples (sessions) for the respective participant. To achieve this, we iteratively removed the channel with the lowest regression coefficient, based on the argument that these channels would have less impact on the overall estimation accuracy compared to the channels with the higher regression coefficients. Table 1 shows the channel count and the corresponding R^2^ that resulted in a statistical power of greater than 0.8. The specific electrode-pairs associated with each channel are presented in Table 2. The difference in contributing channels for each participant highlights the motivation behind an individualized approach in development of models for estimating longitudinal change in motor skill.

**Figure 5:**
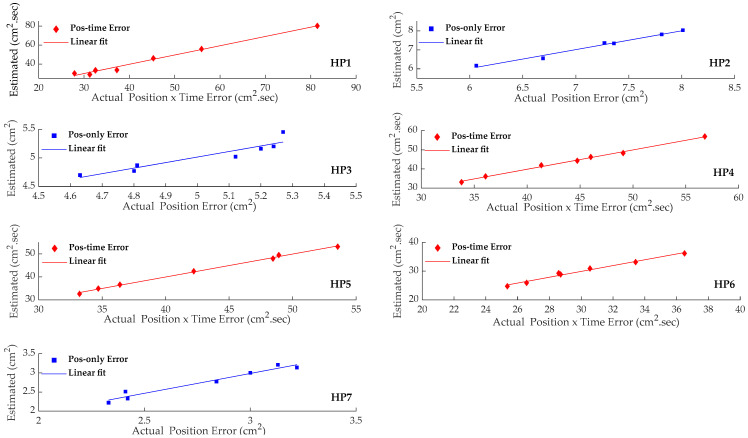
Linear fit of the estimated versus actual tracing performance from a cross-validated leave-one-out approach for each participant. A separate regression model was developed for each participant. The participants’ motor skill was estimated from their corresponding rsFC measures, as identified in Table 1.

Figure 5 shows the results of the cross-validated leave-one-out approach in graphical form. The linear-fit in each graph is generated by the line-of-best-fit using least-squares method. We set the y-intercept to 0 under the assumption that the line should pass through the origin. The mean slope of the Linear-fit was at 0.997. Removing this constraint did not introduce a change in the mean slope of the Linear-fit.

## 4. Discussion

The primary objective in this study was to investigate the accuracy of a proposed individualized method for estimating modest incremental change in motor skill from objective neurophysiological measures. The methodology was expected to address the technical and pragmatic challenges associated with appraisal of motor function to facilitate more frequent assessments. rsFC was selected as the objective neurophysiological measure to allow for the evaluation of wide-ranging network interactions while eliminating the need for execution of physical tasks during assessments. We focused on EEG systems as the measurement modality of choice due to their portability, relative low cost, and because they require a minimal level of the examiners’ expertise and time. These were critical design requirements for a tool that could potentially facilitate more frequent individualized assessments of motor function and subsequent development of personalized intervention strategies. We opted to use computer-based tracing tasks to evaluate spatial error in tracing as an objective measure of motor skill that could be quantified in small incremental changes. PLSC analysis was applied to limit the number of contributing EEG channels for estimating change in skills, followed by PLSR analysis to build a model for estimation. We further reduced the number of predictors (channels) in our estimation model to maintain a statistical power of ≥0.8. The proposed approach resulted in an average estimation accuracy of 98% with a standard deviation of 1.2%. At this level of accuracy in estimating objective measures of behavior, the proposed method may have the potential to provide intermediate valuations of motor function from subjective measures of behavior that inherently have larger margins of error [17,18,21].

PLSC analysis resulted in multiple combinations of behavioral and neurophysiological measures for each participant. The identified channels (electrode-pairs) for each participant were more consistent for rsFC at similar frequency bands and using the same tracing performance option (Pos-only or Pos-time). The latter may indicate the involvement of different brain areas when combining the precision of tracing (Pos-only error) with corresponding speed of tracing (Pos-time error) as a measure of change in motor skill. Similarly, synchronization at different frequency bands may indicate an alternative group of neural networks associated with different aspects of skill improvements [9,36,37,38]. This is further complicated by the individualized nature of learning as can be seen from Table 2. While this study was not focused on cross-participant analysis, it is important for future studies to focus on the localization of most predicting electrode-pairs for a generalized model [18]. This study was focused on evaluating the estimation accuracy of the proposed method rather than identifying the contributing brain areas. Source localization algorithms may have to be applied to better understand the relationship between synchronization frequency and contributing brain areas [9,39]. Prior studies with healthy participants had shown rsFC as a predictor of skill acquisition and the extent of global connectivity as indicators of future motor performance [27,31]. This is consistent with our results with per-training rsFC as predictors of tracing errors. We expected that training-related changes in rsFC would consolidate several hours after the training session [6,40] and would therefore relate to the next pre-training tracing performance. We can see this from the results in Table 1 for five of the participants. The relationship with post-training performance (HP2 and HP4) might be worth further investigation in future studies with larger numbers of participants.

Concerning the measure of tracing performance, we selected the first and last 30 trials to quantify the pre- and post-training skill levels. This was done based on a cursory examination of the variance in tracing performance over the course of all training sessions. Our analysis revealed larger variation in tracing performance for smaller number of trials (<30) during the earlier sessions than later in the training program. We used the ratio of the standard deviation of tracing performance over the corresponding median of those trials (SD-Ratio) for this analysis. This indicated that during earlier sessions when tracing skill was less developed, we needed more trials to get a reasonable estimate of tracing performance. However, as training progressed towards later sessions, the participants could trace more consistently, thereby requiring smaller number of trials. The change in SD-Ratio between 30 and a lower number of trials during these later sessions were relatively small, and as such, we opted to use the first 30 trials as a more stable measure to evaluate the pre-training tracing performance throughout the training program. To maintain consistency, we used the last 30 trials to measure the tracing performance for post-training. It should be noted that in some cases we observed a reduction in tracing performance during the last 30 trials, despite having practiced over the prior 60 trials. This may reflect a confounding factor related to fatigue. A more comprehensive assessment of performance variation over different trial-count selections for pre- and post-training may reveal additional information and is warranted for future studies specific to motor skill improvement.

Our objective for this study did not necessitate a trendline for changes in motor skill, nor a statistically significant change in the acquired skill through out the physical training sessions. Our aim was to investigate the presence of a relationship between rsFC and motor skill (measured through median tracing performance) and whether the relationship could be captured with a regression model to accurately estimate the individuals’ tracing performance from their respective rsFC. We also did not carry out any cross-participant analysis of motor performance, as our objective was to develop an individualized assessment method and not a generalized model to investigate commonality between participants. We speculated that the ability to acquire motor skill was highly individualized and expected to see large differences between participants with respect to the extent and rate of change in skill. The results in Figure 4 are in line with this speculation and support our motivation for the development of individualized models for estimating motor skill.

Inherent in our analysis is the dependence on passage of time. The stable changes in functional connectivity have been attributed to the development of specialized neural circuits for fast and efficient execution of tasks. These changes are not necessarily all associated with an increase in recruitment but also the opposite, indicating that some networks may have become either more efficient or less important for the respective motor skill acquisition [24,41]. We can make similar interpretations about the sign of regression coefficients in our study to indicate an increase or decrease in network recruitment. The longitudinal impact of contributing channels is expected to change as motor skill improves over time. Having a simple and cost-effective tool might facilitate an opportunity to monitor and track the individualized time dependent changes in contributing channels by examining the change in their respective regression coefficients. We expect these changes to be gradual and suggest that new individualized models could be developed based on a rolling reassessment of the rsFC as new longitudinal samples become available. This will also allow for the progressive exclusion of older samples and the associated channels that are less contributing towards the regression model, thereby maintaining the statistical power of the regression analysis. It may also be possible to expedite this temporal change in the regression models by influencing the synchronization levels of the contributing channels through brain stimulation, be it external or through endogenous techniques such as mental imagery and real-time neurofeedback.

## 5. Conclusions

In this study we investigated the accuracy of an individualized PLS processing technique for estimating an objective measure of change in motor skill. We used resting-state EEG to evaluate functional-connectivity indices and position errors from computer-based tracing tasks as a measure of motor skill. We carried out a longitudinal motor skill training program in which seven right-handed healthy participants used a computer mouse with their non-dominant left hand to trace a pattern on a computer screen. Each participant went through six to eight training sessions spread over as many days. We used PLSC to identify the contributing channels specific to each participant and PLSR to develop an individualized model for estimating the longitudinal change in motor skill of the respective participant. Using leave-one-out cross-validation technique, we observed an average root-mean-square estimation error of 1.98%, corresponding to an average estimation accuracy of 98% (standard deviation 1.2%). Considering the pragmatic advantages of using EEG-based resting-state functional connectivity measures for estimating longitudinal change in motor skill, the proposed method shows potential towards an objective and highly accurate motor assessment tool. It may reduce the dependence on the expertise and availability of trained examiners, thereby facilitating more frequent appraisal of the function and development of personalized training programs.

## Figures and Tables

**Table 1:** PLS analysis of the rsFC from pre- or post-training EEG data. PLSC was used to identify the contributing channels and frequency bands that correlated with the tracing performance at *p*-value < 0.05 (uncorrected). The selection of channels was based on robustness of contribution and was evaluated through bootstrapping. PLSR was used to generate the estimation model. The number of channels was further reduced iteratively to obtain a statistical power of 0.8 at α = 0.05 for the estimation model. RMSE is calculated through a cross-validated leave-one-out approach for each model.

Participant	EEG	Freq. Band	Tracing Performance	Channels	R^2^	RMSE (%)
**HP1**	Pre-training	Beta-High (27 Hz)	Single-median Pos-time	5	0.988	4.29
**HP2**	Post-training	Alpha-High (13 Hz)	Dual-median Pos-only	3	0.986	1.06
**HP3**	Pre-training	Beta-Med (21 Hz)	Single-median Pos-only	2	0.855	1.81
**HP4**	Post-training	Beta-Med (21 Hz)	Single-median Pos-time	4	0.995	1.15
**HP5**	Pre-training	Beta-Low (16 Hz)	Dual-median Pos-time	4	0.997	0.98
**HP6**	Pre-training	Beta-Low (16 Hz)	Dual-median Pos-time	4	0.982	1.60
**HP7**	Pre-training	Beta-High (27 Hz)	Single-median Pos-only	3	0.943	2.98

**Table 2:** Contributing channels in the individual regression models for predicting the respective motor skill. Channel-3 for HP3 (Italicized) was excluded from the final model to reduce the number of predictors, thereby increasing the statistical power at the given R^2^.

Participant	Channel-1	Channel-2	Channel-3	Channel-4	Channel-5
**HP1**	T7–CP6	FC1–P4	C4–P3	PZ–OZ	FZ–FC5
**HP2**	F7–FC6	C3–CP5	F3–PO4		
**HP3**	CP1–P3	AF3–FC2	*F8–C4*		
**HP4**	FC2–PO4	FP1–CP1	FZ–T7	AF3–T8	
**HP5**	FP1–T8	C4–PO3	F3–CP1	PZ–PO4	
**HP6**	C4–P3	AF4–OZ	FC5–FC2	F8–CP5	
**HP7**	FZ–T8	F3–P8	C3–PO4		

## Data Availability

The data presented in this study are available on request from the corresponding author. The data are not publicly available due to restrictions for commercial use.
